# Supplementary material for: The Short Form of the Fonseca Anamnestic Index for the Screening of Temporomandibular Disorders: Validity and Reliability in a Spanish-Speaking Population
Source: J Clin Med. 2021 Dec 14;10(24):5858. doi: 10.3390/jcm10245858 (PMC8709097; doi:10.3390/jcm10245858)
Supplement: Supplementary file 1 [file jcm-10-05858-s001.zip › jcm-1493450-supplementary.pdf]

Forma corta del Índice Anamnésico de Fonseca. Versión Española.

Por favor, responda a las 5 preguntas siguientes con NO, A VECES o SI. Marque solo una respuesta para cada pregunta.

	No	A veces	Si
1. ¿Tiene dificultad para abrir la boca?			
2. ¿Tiene dificultad para mover la mandíbula de lado a lado?			
3. ¿Siente fatiga o dolor muscular al masticar?			
4. ¿Tiene dolor de oído o dolor en sus articulaciones temporomandibulares?			
5. ¿Ha notado algún clic en su articulación temporomandibular al masticar o al abrir la boca?			
